# Construct-irrelevant item attributes: a framework to classifying items based on context and referent

**DOI:** 10.3389/fpsyg.2025.1446798

**Published:** 2025-03-12

**Authors:** Wahyu Widhiarso, Rolf Steyer, Andrew Perossa

**Affiliations:** ^1^Institute of Psychology, Friedrich-Schiller University of Jena, Jena, Germany; ^2^Department of Psychology, Universitas Gadjah Mada, Yogyakarta, Indonesia; ^3^Lazaridis School of Business and Economics, Waterloo, ON, Canada

**Keywords:** personality measure, item content, item stems, construct-irrelevant items attributes, item wording

## Abstract

Construct-irrelevant items attributes (CIIAs) are characteristics of psychometric scale items that relate to how item stems are worded, rather than the construct they measure. For instance, an item can be framed from a first-hand (e.g., “How would you describe yourself?”) or second-hand (e.g., “How would others describe you?”) perspective. These attributes meaningfully change the way respondents interpret and answer scale items, so knowing what they are and how they impact data is essential to the construction of valid scales. The present paper serves as a taxonomy of known CIIAs and offers general suggestions on their use. Through this review, we hope to both introduce scale users to the intricacies of item design and offer experienced scale developers a much-needed resource on the types and uses of CIIAs. In doing so, we aim to contribute to the development of more effective, valid scales. We also aim to unify the research literature on item attributes under one taxonomy, to the benefit of scale developers and researchers alike.

## 1 Introduction

Those who develop self-report questionnaires to measure psychological constructs, such as personality, are aware of the inherent complexity of obtaining accurate information about concepts that cannot be directly observed. In addition to the challenges inherent to creating a scale that accurately represents its intended constructs, scale developers must be cognizant of the challenges of wording their item stems effectively (Clark and Watson, 1995). It is not enough for an item to ask the right question, it must be asked in the right way. Asking a question in the right way requires an understanding of how people react to the different contexts with which one can present a question. In this review, we hope to offer this understanding by categorizing and explaining *construct-irrelevant item attributes* (CIIAs).

CIIAs are the aspects of an item that do not relate to its construct (Widhiarso et al., 2019). They pertain to the wording, contextualization, and presentation of items. These aspects, especially item wording, shape how individuals respond to items (DeVellis, 2011). Although the literal meanings of items are drawn from their constructs, people perceive meaning well beyond the intended content (Clark and Watson, 1995). The wording of an item can bias responses, change the implied meaning of questions, and change which aspect of a construct is being evaluated. By identifying, categorizing, and understanding these CIIAs, one can better understand how people will react to the wording of items and how that wording affects the interpretation of the data the scale generates.

The purpose of the present review is to list, explain, and advise on the correct use of CIIAs. Firstly, we hope to introduce researchers who use premade scales to the intricacies of item design. This cognizance should help researchers analyze the strengths and limitations of extant scales more critically, and ensure they select the scales most suited to their methodologies. Further, by explaining why, when, and how scale developers should use CIIAs, we hope to offer guidance to scale developers. Lastly, we aim to unify disparate research on item attributes under one taxonomy, CIIAs. Much research has been conducted on item attributes (Angleitner et al., 1986; Graham et al., 2002; Peabody, 1967), but these attributes have yet to be presented in a unified taxonomy that both scale developers and researchers can use as both a practical reference and a research guide. By combining these findings into a unified list, we hope to simply the task of scale development and encourage future research into CIIAs.

## 2 Basics of constructs, operationalization, and scale design

In psychology and the other behavioral sciences, it is often necessary to measure and analyze abstract, unobservable concepts. Love, happiness, and personality cannot be defined as easily height and weight, let alone quantified. To measure the unmeasurable, researchers represent these abstract ideas as *constructs*. Constructs are theoretical representations of concepts that are constructed in the mind and thus cannot be directly observed, such as happiness (Fried, 2017). Although one cannot weigh happiness, one can develop theories to explain what happiness is, what it looks like, how to categorize its components and variations, and how all these aspects that combine to make someone happy. All the theoretical components that define the essence of an idea form its construct (Fried, 2017). However, one cannot a measure a theory either. To measure a construct in practice one must *operationalize* it.

An operationalization is a tangible, and typically measurable, definition of construct (Ribes-Iñesta, 2003). One might operationalize happiness as the amount of time someone spends smiling per day, the amount of activity in certain parts of their brain, or their answers to a happiness questionnaire. Once a construct is operationalized, one can collect data that can be compared and analyzed (Ribes-Iñesta, 2003). If one asks a group of people how happy they are, it would be difficult to compare the answers in a meaningful way. However, if one gives a group of people a happiness questionnaire comprised of questions based on valid theories, one can compare their scores and make meaningful, valid conclusions about who is happiest. The process of operationalizing theory into useful measures is never perfect (Little et al., 1999). In most fields, both constructs and their operationalizations are constantly being updated, amended, and reimagined.

Operationalizing constructs is the primary purpose of scale construction. In the behavioral sciences, a scale is a measure (usually a self-report questionnaire) designed to determine someone’s level of a construct. Scales have been developed to assess a wide array of traits, behaviors, beliefs, and attitudes (Clark and Watson, 1995). Some scales are taken for enjoyment, such as online personality questionnaires, while others involve high stakes, such as personnel selection tests. When designing the questions, called *items*, for a scale, it is essential to cover every relevant aspect of the target construct (DeVellis, 2011; Loevinger, 1957). For instance, current personality theories posit that there are 5–6 main personality traits, with as many as 8 subcategories for some traits (see DeYoung et al., 2010; Lee et al., 2018; MacCann et al., 2009). Moreover, personality is theoretically complex; comprised of relatively enduring thoughts, behavioral characteristics, and internal dispositions that describe how a person reacts to their environment (Lefton, 2000), as well as relatively enduring patterns of thoughts, feelings, and behaviors that reflect that person’s tendency to respond in certain ways to certain circumstances (Roberts, 2009). Personality scales need to consider thoughts, feelings, behaviors, what respondents say, usually do, and actually do in specific circumstances, all while accounting for the full range of personality traits (Santacreu et al., 2006).

## 3 Beyond constructs—CIIAs and item design

Ensuring that items represent a construct is only the first step in designing items. When people answer items on a scale, all aspects of an item’s wording can influence their responses (Graham et al., 2002). For instance, consider the following items measuring orderliness: “*I try to stay organized*,” “*I wish I were more organized*,” “*I think it’s important for people to stay organized*,” and “*I have never been disorganized in my life*.” All these items appear to assess the same construct. However, it is obvious that one would answer each of them differently. This begs the question: “*Which of these items will offer the most accurate representation of how organized someone is*?” To begin to answer that question, one must understand what makes the items different: *construct-irrelevant item attributes* (CIIAs). It is important to understand which CIIAs are appropriate to the purpose of the scale and the nature of the construct before writing items. For example, different attributes are effective in personality scales developed for personnel selection than those developed for research, even though they measure the same constructs. Items developed to measure extraversion might have different characteristics than items that assess agreeableness because of how differently those traits manifest in people’s behavior (John and Robins, 1993). With a clear understanding of the specific purpose of the scale being developed, CIIAs can be strategically used or avoided to maximize a scale’s validity and reliability.

Table 1 shows that CIIAs, which measure related but indirect aspects of the trait, can represent the same facet. For example, instead of directly stating preparedness as a construct-relevant attribute, CIIAs help the item writer to express preparedness into behaviors or opinions, such as “*I make checklists to ensure I am prepared*” (behavior) or “*I believe that being prepared is a key to success*” (opinion). These items provide additional perspectives on the facet but may include elements not central to the core construct, offering a broader or contextualized understanding of the trait of interest.

**Table 1:** Example of creating items using construct-irrelevant item attributes.

Facet	Content	Construct irrelevant item attribute
Self discipline	Always prepared	I believe that being prepared is a key of success (opinion)
		I make checklists to ensure I am prepared (behavior)
	Initiate tasks promptly	I often initiate tasks promptly to maintain momentum (continuous)
		I take action on tasks as soon as possible (non continuous)
Orderliness	Like order	I prefer to create detailed schedules to organize my activities(1nd hand source)
		My friend noted that I am pleasure following a detailed schedule(2nd hand source)
	Stict to the plan	I perform better when strictly adhere to my plans rather than improvising (discrete)
		I prefer to stick to my plan to avoid distractions (non discrete)

The CIIA is not a novel concept. Most trait theories propose that the evaluation of traits or behaviors should be conducted in diverse contexts, encompassing trait-relevant activities together with their intensity, frequency, and length (Amelang and Borkenau, 1986). The assertion is supported by other experts who state that employing multiple-act criteria, which involves evaluating actions across several instances, is more effective than simply repeating similar activity (Gifford, 1982). Alternatively, constructs may be assessed according to the diversity of referents and the range of contexts sampled. Moskowitz (1982) use the word “referents” to signify the measurement of a construct through the assessment of referent diversity and the range of sampling instances. An instance of a referent he presents is settings and occasions, which is sample of types of CIIA.

Researchers have divided the item stems of psychological measures into several categories. These categories are called *category system of item-trait relations* (Lennertz, 1973), *item characteristics* (Angleitner et al., 1986), *item content domain* (Werner and Pervin, 1986), *item contextualization* (Schmit et al., 1995) and *item attribute* (Graham et al., 2002; Mael, 1991). All of those have the same purpose of supporting more systematic construction of item pools because this process usually employs an idiosyncratic process that is not reproducible (Loevinger, 1957). The basis used by researchers to develop the concept varies. Lennertz (1973) classification was developed based on indirect association of items and personality traits whereas Angleitner et al. (1986). start their concept by defining several types of potential verbal manifestations of traits and classify surface structures of questionnaire items. Both ideas support Loevinger’s (1957) recommendations to ensure that the item pool is selected to include all potential contents that may constitute the target trait, in alignment with all recognized alternative theories of the trait.

Construct-irrelevant variance (CIV) and construct-irrelevant item attributes (CIIAs) are distinct concepts but have common similarity. Both concepts are non-essential to the construct of interest, they work at different levels and have varying implications for the validity of measurement. CIIAs work on item levels to cover any possible construct representations for generating item pool. CIIAs serve as a framework for formulating construct into items in questionnaires. For instance, a domain or dimension of the construct of interest can be represented by two factual or non-factual items. Factual items are more likely to assess behavior while non-factual items assess opinion even though both items measure similar construct. In contrast, construct irrelevant variance (CIV), also known as construct contamination, refers to variation emerging from factors unrelated to the construct of interest. CIV poses a major threat to the validity of test interpretations as it introduces systematic errors that can distort the true information.

## 4 Types of construct-irrelevant items attributes

### 4.1 Extremity

*Extreme* items contain absolute descriptors such as *always* or *never*. Most of the research on extreme items concludes that scale developers should usually avoid using them (e.g., Nunnally and Bernstein, 1984). In addition to recommending against extreme wording (e.g., “*My friends are always brilliant*”) researchers also recommend that scale developers avoid the use of neutral language (e.g., “*My friends are all right*”), as item stems should have favorable and unfavorable poles (Dörnyei and Taguchi, 2009). Clark and Watson (1995) recommend avoiding extreme wording because respondents may be less likely to endorse such items. However, as extreme wording can give useful information about the response process, several scales employ these types of items. For example, many items of the Marlowe-Crowne Social Desirability Scale contain extreme wording (e.g., “*I always try to practice what I preach*”). Another popular scale, The Minnesota Multiphasic Personality scales (MMPI) uses extreme wording to detect dishonesty in a clinical context. Although the purpose and setting are different, several scales designed for use in organizational settings also incorporate extreme item wording. Nye et al. (2010) found that extreme items may be helpful for some purposes. Theoretically, people tend to agree with statements that that they feel accurately represent themselves and disagree with statements that they do not feel represent themselves. Asking respondents whether an extreme level of a trait represents them means that only those with correspondingly high levels of the measured trait will tend to agree.

### 4.2 Target, action, context, and time (TACT) orientation

Ajzen and Fishbein (1977) found that attitudes better predict a participant’s future behavior when those attitudes are measured at the same level of specificity as the target behavior. This observation is known as the *principle of compatibility*. For scale developers, this means that self-report items aimed at predicting behavior should be as specific as the target behavior. For instance, the item “*I intend to register as an organ donor this year*” will predict whether people register this year much more accurately than the item “*I support organ donation*” (Demir and Kumkale, 2013). To adhere to this principle, Ajzen and Fishbein (1977) suggest that such items should contain a *target* (i.e., what the behavior is directed toward), *action* (i.e., actual behavior), *context* (i.e., the situational context surrounding the behavior), and *time* (i.e., when the behavior takes place). These four aspects—target, action, context, and time—are abbreviated as TACT. Items that include these four aspects, thereby fulfilling the principle of compatibility, are more likely to predict whether respondents engage in the target behavior, which is essential to any behavioral scale.

As such, an item such as “*I intend to apply for a job*” will not predict job choice as well as the item “*I intend to apply to company X this fall*” (Ployhart and Ehrhart, 2003). The first item does not meet all TACT criteria, while the second does. The separate TACT elements are also exemplified in the following sample item: “*All the work I did in the office within the last month has been consistent with the office plan*.” In this example, the *target* element is “the office plan,” the action is “all the work I did,” the context is “in the office,” and the time element is “within the last month.” TACT posits that people’s attitudes, beliefs, and behavioral intentions work together to eventually form people’s behaviors. The TACT framework is especially useful for the development of TPB-inspired scales (Conner and Sparks, 1996). However, the TACT framework is not frequently used in other scales, such as personality inventories, since these scales often assess not only observable behaviors, but also cognitive and affective aspects of personality.

### 4.3 Domain specificity

Patton (2001) proposes seven domains that can be assessed using personality scales: behavior, opinions, values, feelings, knowledge, sensory, and background. These domains are usually used in interviews but are also relevant to item stems in personality scales. Four of these domains are usually used in the development of self-report assessments. The first is behavior; items in this category reflect what individuals actually do. Behavior may vary by intensity (e.g., “*I do my work very carefully*”), expectations (e.g., “*I will not waste time when working*”), or motivation (e.g., “*I do my work carefully so as to not disappoint my boss”*) (Patton, 2001). The second domain, opinions, assesses individuals’ opinions, values, or views on a topic. Such items are often based solely on personal judgment and aim to understand the cognitive and interpretive processes by which individuals arrive at opinions, judgments, and values. Responses to these items tell researchers what respondents think about some experience or issue and give information about respondents’ goals, intentions, preferences, and expectations.

The third domain, feelings, contains items aimed at eliciting emotional responses to objects. Adjectives such as “anxious,” “happy,” “afraid,” “intimidated,” or “confident” are therefore often used in these items’ stems. Opinions and feelings are sometimes confused as both domains often require that an object be appraised or evaluated. To distinguish between these two domains, one should note whether the item refers to emotional reactions. Items in the feeling domain typically mention discrete emotions, such as “happy” or “irritating,” whereas items in the opinion domain do not. The fourth domain, background, assesses to what extent individuals’ personal backgrounds might correlate with certain attitudes, skills, or personality traits. Items in this domain may inquire about any aspect of one’s background: gender, race, ethnicity, age, educational level, medical problems—even golf handicap or the name of a pet. These items may offer a constrained set of choices or blank spaces to fill in the information. Since the purpose of these items is to gather factual information, individuals must provide accurate information and not bias their responses.

### 4.4 Willingness

Situational judgment tests (SJTs) items can be divided into two types: what one *would do* and what one *should do* (Ployhart and Ehrhart, 2003). This framework can be applied to items of questionnaire. *Would-do items* are designed to assess how the respondent would react to and behave in a hypothetical situation. In contrast, *should-do items* correspond more strongly to opinion rather than intention to perform, and may therefore not strongly relate to actual job performance. Asking respondents what they would do is more predictive than what they should do because would-do items adhere to the principle of compatibility (Ployhart and Ehrhart, 2003). The use of questions of this type is debated. Several researchers suggest avoiding the use of hypothetical questions altogether (Walther et al., 2007). Instead of asking hypothetical questions, they argue that scales should ask about actual behavior.

Some researchers believe that it is difficult for respondents to answer questions about imaginary situations, as they relate to circumstances they may have never experienced. Hypothetical questions are often asked to get more insight into attitudes and opinions about certain issues, but little is known about the processes in the respondent’s mind that lead him or her to give particular answers to such questions (Bethlehem, 2009). The difference between would-do and should-do items is relevant to the process of constructing items for personality scales. Asking a hypothetical question prompts a hypothetical answer about what might have happened in respondents’ lives under different circumstances, or what they might do if confronted with an unusual and difficult task (Converse and Presser, 1986). Would-do items meet the criteria for hypothetical action, including the attribute of being *time-framed* (e.g., pertaining to future behavior).

### 4.5 Factuality

*Factual items* ask individuals to report information about facts that might refer to explicit behavior. Since the information being asked for is factual, the true answer could always be determined by some means other than asking the respondent or using theory (Bethlehem, 2009). Factual items may rely on the respondent’s memories, such as when items ask about the frequency of individual behavior (e.g., how often one interacts with customers). Bryman and Bell (2007) divide factual items into three categories: personal factual questions, factual questions about others, and informant factual questions. In contrast, *nonfactual items* inquire about attitudes and opinions. An opinion usually reflects one’s views on a specific topic, such as what makes a good employee or an effective decision. Attitudes are more general and are comprised of one’s conscious and unconscious feelings, opinions, and reaction tendencies toward something (Gawronski et al., 2006). Individual opinions and attitudes should not be labeled as inherently good or bad because they refer to subjective states. As with factual questions, nonfactual questions can be divided into three categories: questions about beliefs, questions about normative standards and values, and questions about knowledge (Bryman and Bell, 2007).

Understanding the differentiation between factual and nonfactual items is important not only for open-ended questionnaires, but also for personality scales, as personality scales can assess factual or nonfactual indicators of the assessed constructs (Back et al., 2009; Riggio and Riggio, 2001). Factual aspects manifest as behaviors while nonfactual aspects manifest as thoughts. Actual behavior exists independently of any person’s report of the behavior, and a self-report is true or valid to the extent that it corresponds to the actual behavior. Factual items can also be verified by follow-up investigations. For example, an employer many conduct an additional assessment (e.g., interview) to investigate the validity of the factual responses provided on a questionnaire. Waltz et al. (2010) describe how the validity of factual responses can be verified using external sources of information (e.g., clinical records) or consistency checks within the questionnaire itself, whereby the same information is requested in more than one way. Many personality scales include items that inquire about factual information in terms of behavior or explicit indicators. Examples include the conscientiousness item “*I strive for excellence in everything I do*” in NEO-PIR (Costa and McCrae, 1989) and the depression item “*I cry often*” in CES-D Scale (Radloff, 1977).

### 4.6 Directionality

Most personality scales include a combination of positively and negatively worded items to reduce bias (Spector, 1992). For instance, a scale designed to measure self-esteem will have some items written in a positive or favorable direction (e.g., “*I feel that I have a number of good qualities*”) and others written in an unfavorable or negative direction (e.g., “*I feel I do not have much to be proud of*”). A person with high self-esteem should endorse positively worded items and reject negatively worded items. By varying the item direction, biased responses produced by respondents’ response styles and tendencies can be reduced. One such tendency is *acquiescence*, the tendency for respondents to agree or disagree with all items, regardless of content (Spector, 1992). Combining positively and negatively worded items in personality scales— typically using approximately equal numbers of positively and negatively worded items—is a common strategy to reduce acquiescence bias and detect careless responding. Marsh (1996) found that combining positively and negatively worded items generates a factor structure for self-esteem that is different from the original structure. Marsh (1996) observed factor variance that did not correspond to general self-esteem but was instead associated primarily with the positively and negatively worded items themselves. Interpreting such a structure is difficult, because being positively or negatively worded represents the method rather than the trait being measured. This phenomenon is consistent with other measurements that mix positively and negatively worded items, as factor analyses frequently reveal different factor structures for each direction (Biderman et al., 2011). However, semantic polarization of the items can be handled using an appropriate measurement model (Vautier and Pohl, 2009).

### 4.7 Frame of reference: context specificity and time frame

Context specificity is a frame of reference concerning the specificity or generalizability of the context surrounding and item. Context specificity can be divided into two general levels: global and contextual. Global items are non-contextual and generalizable to any situation, such as “*I pay attention to detail.”* Contextual items specify a given context, such as “*I pay attention to details at work.”* The differences between these categories are highly relevant to the construction of personality scales. Global items allow you to assess attitudes that are cross-situationally consistent. However, in order to assess situationally specific personality constructs, a context must be specified (Schmit et al., 1995). Items on personality scales generally inquire about behaviors that can be generalized across situations. Individuals are assumed to follow patterns of behavior or thought in their responses to the items. For example, individuals respond to items assessing general conscientiousness in relation to their self-perceived conscientiousness, which reflects their general tendency toward conscientiousness behavior stable across different situations.

Alternatively, personality scales can rely on general assumptions about individual experiences, developing assessments based on context-specific items that assume people tend to have fairly stable patterns of behavior (contingent on specific situational conditions; Wright and Mischel, 1987). Since the relationship between personality and behavior may be limited to a specific range of situations, item stems should explicitly specify the situation or location (e.g., work or school) when assessing behavior. The uses of context-specific items not only adheres to the principle of compatibility, but is also supported by the cognitive-affective system theory of personality (Mischel and Shoda, 1995). According to this theory, it is only when situations elicit psychologically similar patterns between cues and demands that a researcher can assume individual responses to be cross-situationally consistent. The implication is that clarifying the context or frame of reference in the item can improve response accuracy when individuals are asked to describe themselves (Lievens et al., 2008). Global item stems are generic, thereby remaining open to different interpretations by respondents. Since the context for the assessed behavior is not constrained, respondents are unrestricted in their ability to associate the behavior with different contexts and situations.

Another relevant frame of reference is time frame. That is, whether the item pertains to the respondent’s present, past, or future experiences. For example, researchers could ask respondents what they are doing now, what they have done in the past, or what they plan to do in the future (Patton, 2001). To assess a trait, it may be helpful to inquire about how multiple indicators manifest differently in the present, past, or future. Personality scales are sometimes developed with this consideration, as their items sometimes inquire about past behavior in order to predict future performance outcomes (Russell, 1990). If respondents associate a scale’s items with different frames of reference (e.g., with regard to time or context), responses will become inconsistent, which can be detrimental the scales validity and reliability. For example, if one respondent thinks that an item is assessing behavior in the work setting, they may report behavior that represents a good worker. If another individual assumes that the same item is associated with their everyday life outside the work setting, they may report their behavior differently, appearing to be a less desirable worker. Setting the frame of reference explicitly in the item stems is therefore essential to reducing between-person inconsistency.

### 4.8 Transparency

Transparency refers to how obvious it is to the respondent what construct an item is intended to measure (Zickar and Drasgow, 1996). When an item is less transparent, or *subtle*, it is not readily apparent what the item measures. An example of a subtle item on the MMPI is “*I enjoy detective stories.*” This item appears to assess hobby or activity preference but could also assess indicate interest in investigating unsolved problems. When an item is more transparent, or *obvious*, most respondents will recognize what construct the item measures (Zickar and Drasgow, 1996). For instance, “*I am conscientious*” is an extremely obvious item that measures conscientiousness. Scale developers usually employ subtle items to prevent individuals from faking their responses. When individuals have a higher intention to fake their responses to items assessing psychopathology on the MMPI, their scores on subtle items tend to be lower than those who have lower intentions to fake (Dannenbaum and Lanyon, 1993). In contrast, obvious items have high face validity because the construct is readily apparent in the item. Their content therefore exhibits a straightforward, unambiguous relationship to the construct of interest (Bagby et al., 1998).

The main characteristic of subtle items is that they ask individuals to report an event or experience that is not obviously associated with the construct. Subtle items might ask about individuals’ hobbies, work preferences, or desire to participate in activities and avoid asking about things that are clearly opposites like good and bad. Subtle items therefore rarely contain adjectives and typically refer to experiences that most people can relate to. Researchers are most likely to include subtle items on personality scales when they are using external methods of scale or test construction. The external method of test construction is often non-theoretical, with a focus on identifying items that empirically differentiate two or more criterion groups (Hough et al., 1990). Individual endorsements of such subtle items are therefore tailored to the criterion groups. For example, if it is found that most individuals with high levels of conscientiousness enjoy detective stories, then individuals who endorse the item “*I enjoy detective stories*” will get higher scores in conscientiousness than who do not. The ability to create valid, subtle items is one of the benefits of the external test construction strategy (Bagby et al., 1998).

Results regarding the performance of subtle items have thus far been mixed, with some researchers finding subtle items necessary and others reluctant to use them (Dannenbaum and Lanyon, 1993). The scoring procedures of subtle items have also been found to be ineffective at discriminating between individual attributes when faking occurs. Dannenbaum and Lanyon (1993) found that when individuals attempted to fake their responses to MMPI in a pathological manner, they tended to endorse the subtle items differently than they endorsed items assessing psychopathy. Subtle items were found have lower item discrimination parameter estimates, and an interaction was found between subtle items and social desirability. Items that are less subtle or higher in social desirability tend to have lower location parameters (Zickar and Ury, 2002). As such, although many personality scale developers recommend avoiding subtle items, some scale developers prefer subtle items over obvious items because subtle items appear more resistant to motivated faking and socially desirable responding (Zickar and Ury, 2002).

### 4.9 Descriptiveness

When making judgments, people often communicate their opinions using trait terms such as “good,” “bad,” or “happy.” These trait terms can be sorted into two broad groups: descriptive and evaluative (Peabody, 1967). Descriptive trait terms are more factual, such “wealthy,” whereas evaluative trait terms pass judgment, such as “rude.” Evaluative terms judge something to be either good or bad, with each term existing in opposite pairs (e.g., “kind and unkind”). Peabody (1967) posits that any such trait can be divided into four groups. To give Goldberg’s (1993) example, the four traits of generous, thrifty, extravagant, and stingy represent a combination of the evaluative aspect (desirable vs. undesirable) and the descriptive aspect (spending lots of money vs. spending little money).

Applying this taxonomy to personality scales shows that personality scale items can have two types of wording. The first type is items used to communicate personality judgments, which entail some degree of either approval or disapproval. These items represent individual judgments, and typically involve an evaluation process. This process results in a choice between two polar meanings, such as liking–disliking or good–bad. The second type is items that remain conceptually independent of the evaluative aspect by holding the evaluation aspect constant (Saucier et al., 2001). Items of the first type include the evaluative aspect (e.g., “I do well in my current position”) while the latter includes only descriptive or non-evaluative aspects (e.g., “I am happy with my job”).

Items assessing the evaluative aspect are thought to be more sensitive to faking, as they imply that something is considered good or virtuous. Bäckström et al. (2009) argue that items with evaluative aspects allow response distortion, as items with an obvious valence (e.g., positive or negative) make the socially desirable response more readily apparent. Thus, respondents with a strong desire for social desirability will endorse obviously positive items more strongly than subjects who are lower in social desirability. Bäckström et al. (2009) noted that item stems that encompass evaluative elements tend to be highly correlated, because individuals with a strong desire for social desirability will uniformly endorse them. For example, items in the NEO Five Factor Personality Inventory (NEO-FFI), which measures five different personality factors, may have a high correlation among themselves, since many items encompass evaluative elements. As a result, correlation among the five personality factors of this inventory is high, although these factors should have low intercorrelations (Bäckström et al., 2009).

There are two models for personality scales regarding the implementation of evaluative and descriptive aspects: confounded and unconfounded (Saucier et al., 2001). In the confounded model, scales use items that contain both descriptive and evaluative aspects (e.g., “*My work is bad because I rarely work persistently*”). In the unconfounded model, items with evaluative and descriptive aspects are clearly separated. Items in the unconfounded model are preferable to items that include evaluative aspects as they reduce the effects of social desirability bias. Descriptive items are important since they assess specific domains of the attribute being measured. For example, (Brinthaupt and Erwin, 1992) suggest that descriptive aspects represent self-concept while evaluative aspects represent self-esteem; it is therefore not necessary that these aspects be strictly separated. Applying this logic, both aspects can be included in self-report scale, but evaluative content should be less frequent than descriptive content.

In light of these findings, several personality scale developers have emphasized the descriptive element by reducing the evaluative aspect, as Jackson (1967) did when developing items for the Personality Research Form (PRF). Edwards (1957) also employ item with evaluative referent when developing the Social Desirability Scale (SDS). Both scales were designed to measure social desirability. Bäckström and Björklund (2014) also support this approach through their method of *evaluative neutralization*. This method separates the evaluative aspects of items in a self-report by rephrasing positive items to appear less positive and negative items to appear less negative, thereby making all scale items relatively more neutral. For example, the evaluatively loaded item of “*I get upset easily*” can be reframed as “*I sometimes react strongly to things that happen.*” Or, the item “*I feel little concern for others*” can be reframed as “*I believe it is better if everyone cares for himself or herself*.” Another example of evaluative neutralization implementation can be found in Bäckström et al. (2011) for two items that measure conscientiousness. The item “I make plans and stick to them” is desirable because it focuses on the ability to guide oneself in the direction of one’s goal. In contrast, the item “I avoid departing from a plan once I have made one” focuses on the unwillingness to change plans. Respondents with high conscientiousness will endorse both items, but respondents with high social desirability will endorse the first item since it describes an attractive trait. According to the authors, separating the evaluative and descriptive aspects of items is similar to contrasting items that measure the same construct but differ in evaluative content (Bäckström and Björklund, 2014). Evaluative and descriptive aspect differentiation is one approach for constructing item stems on personality scales. This approach does not focus on the content of the trait being measured, but rather on content representation. Although two items may measure similar content, if either their evaluative or descriptive aspect is reduced, the item stems are viewed differently by the respondent.

### 4.10 Verifiability

Verifiability refers to whether the information an item asks for could be corroborated by independent sources. Mael (1991) likens verifiable items to archival data (e.g., work records), as they can be objectively verified. Non-verifiable items pertain to the respondent’s thoughts, feelings, or behavior in ways that can only be known by the respondent. Though verifiability was proposed as a necessity for biodata items (Mael, 1991), personality scales can also utilize this feature to assess a broad range of psychological constructs such as subjective internal states, behavioral responses, and hypothetical reactions (Hough et al., 1990). Although much of this psychological information is only known to the individual, general traits emerge that may be possible to verify. Personality scale item stems have different degrees of verifiability (Fernandez-Ballesteros and Marquez, 2003), an important consideration for examining response validity and accuracy.

The verifiability of item attributes is a primary classification that can be applied to other attributes that are more specific to the content. For example, items that can be categorized as first-hand or second-hand within the attribute *source of information* can also be defined as verifiable or unverifiable; first-hand items are considered unverifiable since only individuals know the truth of their responses. Items that can be categorized as subjective or objective within the attribute *judgment* can also be defined as verifiable or unverifiable. Further, objective items (which pertain to overt behavior) are verifiable, whereas subjective items are not.

### 4.11 Continuity

Continuity refers to whether the range of possible item responses or item lay on an obvious continuum (Doll, 1971). According to Graham et al. (2002), the item anchors of continuous items represent a clear continuum for the given construct, representing different degrees of the attribute being measured. For example, item stems with response options expressing varying degrees of agreement (agree–disagree) or behavior frequency (always–never) are considered continuous items. In contrast, forced-choice items, for which the response options are one of two or more different traits with equal endorsement attractiveness, are considered non-continuous items (e.g., “creative or dedicated”). However, the attribute of continuity also applies to the content of item stems. Continuous item stems usually use terms that represent qualitative degree, such as “*moderately*” or “*sometimes*.” Used as modifiers, these terms may increase or decrease the intensity of the trait being assessed. The item “*I rarely handle my work in a disorganized fashion*” is continuous, as it follows a continuum of frequency of action occurrence. In contrast, non-continuous item stems do not use modifiers. Instead, they assess single traits without defining intensity. One example of such an item is “I do my job in a professional manner.”

### 4.12 Internality

Mael (1991) proposed the attribute of *internality* for items on biodata measures. This attribute reflects whether the behavior that the item assesses refers to overt (external/manifest) or covert (internal) behavior. For biodata measures, external items assess expressed actions (e.g., personal experience skipping a day of school), while internal items refer to attitudes, opinions, or emotional reactions (e.g., one’s attitude toward people who skip school). In addition to being used with biodata items, the internality attribute can also be applied to personality scales. Bellack and Hersen (1977) described several types of information that can be gathered by items on self-reports. The first type of information is from motor, physiological, or cognitive-behavioral response systems (e.g., “I have problems with my muscles” or “*I have to think hard when working with numbers*”). The second type of information concerns individuals’ subjective experience or evaluation of these motor, physiological, or cognitive behavioral response systems (e.g., “*I am a heavy smoker*” or “*I often feel anxious*”). The third type of information concerns stimuli or the way individuals perceive these stimuli (e.g., “I often feel threatened when approaching a deadline”). This categorization suggests that there are two types of self-report items regarding response to stimuli: external events (e.g., behavioral response) and internal events (e.g., physiological responses). Hence, the internality attribute emphasizes the type of expression assessed by items, contrasting individuals’ internal and external events.

### 4.13 Controllability

Individuals can control their involvement in some activities (e.g., walking or speaking) but cannot control other activities (e.g., growing or dying). The item attribute of *controllability* emphasizes this distinction by assessing whether respondents can modify their responses to meet their needs. This distinction concerns the events which correspond to the performed actions and background events (Girotto et al., 1991). From the biodata perspective, events and experiences may not only describe individuals’ underlying dispositions, but also determine subsequent behavior and potential modifiers of dispositional responses in the future. This distinction implies a better understanding of the type of items that refer to uncontrollable behavior (Mael, 1991). The types of behavior measured by items may range from controllable to uncontrollable actions.

A controllable action needs external stimuli to occur, while non-controllable actions are autonomous and do not always require external stimuli. For example, the item “*I attempt to finish my work on time*” is controllable, since the individual can choose to try to finish their work on time. Essentially, controllable events work like a switch, which can be turned on or off, whereas non-controllable events cannot be prevented from happening (Leduc et al., 2009). In addition to classifying behavior as controllable or uncontrollable, behavior can be classified as *implicit* or *explicit*. According to Moors et al. (2010), implicit (often referred to as *automatic*) behavior is unconscious, unintentional, or uncontrollable, while explicit behavior is more controllable, deliberate, intentional, and conscious. Scale developers may then select controllable items when they want to focus on intentional processes. Individuals will, however, report the assessed attribute with varying degrees of accuracy and may distort their responses. Scale developers may select uncontrollable items when they wish to assess an attribute that cannot be controlled by respondents.

### 4.14 Source of information

The extent to which respondents are perceived to embody certain personality traits can change based on whether the source of information is perceived or received. The most popular example of this categorization is the self-report. Self-reports can assess one’s own evaluation of the attribute of interest, or the evaluation made by others. Likewise, when personality scales inquire about individuals, the provided information can be obtained from either individuals (first-hand source) or others (second-hand source). Distinguishing between items that use these two different sources of information can describe the extent to which items ask about personal, direct observation or others’ evaluations (Becker and Colquitt, 1992). Distinguishing between items that use these two different sources of information can indicate whether items ask about self-evaluation or others’ evaluation. Lieberman et al. (2001) refers to items that inquire about others’ evaluations as *externalized self-perception*, meaning how one thinks one is seen in the eyes of others. Several self-report measures include item stems that rely on second-hand information, such as “*Some of my friends think I’m a hothead*” in the Aggression Questionnaire (Buss and Perry, 1992) and “*My friends see me as a clown*” in the Defense Style Questionnaire (Andrews et al., 1993). Using items that request second-hand information assumes that there is reasonable congruence between individuals’ self-evaluations and how they perceive others to evaluate them. This occurs because self-perceptions result from the internalization of the perceived views of others. Recent findings suggest that there is congruence between not only the self and the perceived views of others, but also the self and others’ actual evaluation (Hensarling and del Carmen, 2002). However, the connection between self-perception and the perceived evaluation of others cannot be generalized for a broad range of personal attributes.

### 4.15 Objectivity

Information obtained from self-report assessments is subjective; items must fulfill stringent criteria to qualify as objective measurement. Objective items inquire about factual evidence of individual attributes, while subjective items inquire about opinions or feelings, rather than facts (Jackson, 2009). Since self-report always involves subjective judgments, it usually considered a subjective measurement. In contrast, objective measurements involve little individual judgment in the collection and processing of information (McDowell, 2006). The definition of objective items above is in line with the definition provided by Morrow and Jackson (2000), who argue that items that require respondents to select one or more given responses can be scored with minimal subjective judgment, and thus can be categorized as objective items. Morrow and Jackson (2000) focused on how different scoring procedures, such as true/false, matching, and multiple-choice questions fulfill the requirement of objectivity.

The distinction between objective and subjective items can also be explained by the distinction between whether an event occurred (objective) and how the individual perceived the event (subjective). Using this definition, an item that provides a statement about one’s experience is objective because an experience is an event that actually occurred. Brucks (1985) also supports this idea, describing three categories of consumer product class knowledge: subjective knowledge (the individual’s perception of how much she or her knows), objective knowledge (what an individual actually knows), and prior experience (amount of individual experience). The categorization of objective and subjective knowledge is problematic when individuals cannot accurately perceive how much they actually do and know. Items that measure objective knowledge are not always entirely objective. The accuracy of objective items may depend to some extent on the momentary cognitive and affective state of the respondent. It can therefore be inferred that items asking about past action or assessing knowledge are classified as objective statements.

## 5 Conclusion

Deriving trait indicators into several manifestations in personality scale construction can employ CIIAs as a framework in writing items. CIIAs would not only provide guidance for generating item pools but also explore which item types are most relevant to the traits under investigation. Table 2 demonstrates how items in personality surveys can be rephrased with varying referents and behavioral expressions while continuing to evaluate the same construct of interest. CIIAs classify items according to various attributes, such as verifiability (e.g., “My experiences demonstrate that I…” is deemed more verifiable compared to “I am a committed person,” which is less verifiable), source of information (e.g., first-hand assertions like “I am a person who works diligently” versus second-hand evaluations like “My supervisor rates me as a diligent worker”), and domain specificity (e.g., particular actions such as “I do my work very carefully” in contrast to general characteristics like “I am a disciplined person”). This categorization demonstrates how personality traits can be expressed in various ways, allowing for flexibility in item construction while capturing different aspects of the same underlying construct.

**Table 2:** Example of item wording using several types of CIIA.

Type	Value	
Verifiability	More verifiable	Less verifiable
	My experiences demonstrate that I …. It is not difficult to prove that I am ….	I have been able to overcome boredom at work. I am a committed person.
Source of information	1st hand information	2nd hand information
	I am a person who works diligently. My skills are above average.	My supervisor rates me as a diligent worker I am as healthy as anybody I know
Domain specificity	Domain Specific	Domain Non-specific
	I do my work very carefully (action). I love regularity (feeling).	I am essentially a modest person. I am a disciplined person.
Transparency	Transparent	Subtle
	I am conscientious. I am full of energy.	I enjoy detective stories. I like exercising regularly.
Direction	Positive	Negative
	I like order. I like to tidy up.	I leave a mess in my room. I am not bothered by disorder.
Time frame	Present	Future (Hypothetical)
	I complete projects according to plan. I do my work carefully.	I try complete projects according to plan. I try to do my work carefully.
Discreteness	Non-discrete	Discrete
	I am a disciplined worker. My achievements result from my perseverance.	I am better described as a disciplined person than as a tolerant one. My achievement comes from my perseverance, rather than friends’ support.
Continuity	Non-continuous	Continuous
	I do my job in a professional manner. I handle my work in an organized fashion.	Sometimes I do my job in a professional manner. I rarely handle my work in a disorganized fashion.
Internal-external	Internal	External
	I focus on my job even my mood is poor. I like to go into the detail of my tasks.	Mood should not affect an individual’s effort when finishing a job. People should pay attention to the details.
Context specificity	Defined	Undefined
	At work, I often plan things in advance. To date, I finish my work on time	I often plan things in advance. I finish my work on time.

When implementing the use of CIIAs in writing items, some considerations should be taken into account. First, there is a relationship between one and other types of CIIA. Some types of CIIAs have the same reference, and one type is a consequence of the other type. For example, items referring to behavior will clearly be more objective than those that refer to feelings. As a consequence, this item will be more verifiable. Second, certain types of CIIAs align with a construct by embodying its typical characteristics. For example, the neuroticism scale usually employs more items describing covert behavior, whereas extraversion employs items referring to overt reactions in a scale (Angleitner et al., 1986). Third, the use of CIIAs in scale development can be tailored to the purpose of measurement. For example, the development of measurement instruments for selection purposes can consider types of CIIAs that enhance item resistance to response distortion. Widhiarso et al. (2019) found that some types of CIIAs can reduce the appearance of faking on psychological scales when applied in work situations. The use of different types CIIAs can produce items with different levels of difficulty but still have high discrimination. This is because the use of different contexts and referents affects the intensity or degree of item to represents level of construct being measured. This condition supports the development of measure in Computer Adaptive Testing (CAT) where the items in the item bank or pool should have different levels of difficulty.

Though this listing of these CIIAs is, presently, comprehensive, our review of the research surrounding them is not. To use this review as a guide, one must highly be cognizant of all of the needs and limitations of one’s scales, the environment that scale will be administered, and how other aspects of one’s research design or practical context may affect respondents’ mindsets. We examine the effect of construct-irrelevant item attributes (CIIA) on item parameters (Widhiarso and Putra, 2025). Seven CIIAs along with their two opposite attribute values that describe the degree of items that adhere to the respective attribute were proposed. The results of the analyses found small differences in item discrimination and item difficulty between attribute values in seven CIIAs. This study show that different item wordings have the least impact on the psychometric properties. Our findings suggest that the similar performance of items with different wording may be due to the fact that construct-relevant item attributes are more likely to explain the variation than irrelevant item attributes. The analysis suggests that even though two items employee different attribute, both items still account for most of the true score variance. The correlation of item score between two items that refer to two different attributes obtain correlation between 0.538 (discreteness) to 0.921 (context specificity). This means that the item scores for both items are very closely linked. Other studies have found that using items in questionnaires with a wide range of trait referents does not interfere with the psychometric properties of the items or tests, and even some studies have found that using a variety of CIIAs can improve measurement quality (Jaccard, 1974; Nye et al., 2010). However, some other studies have found that certain categories of CIIAs, such as negatively worded items, can reduce the reliability of the measurement (Zeng et al., 2024).

Standard guidelines for scale development emphasize the importance of defining the construct appropriately, selecting domains to capture, and providing items that address a wide range of trait manifestations. CIIAs can be used to enrich the item pool by involving context and referents in the item stem. However, certain CIIAs may be relevant to the thing being measured, and presence method variances can threaten the validity of the measure. For instance, items representing covert reactions, such as “I think a lot about myself,” can be attributes relevant to measuring introversion (Angleitner et al., 1986). Similarly, negatively worded negatives can be attributes that are relevant to the measurement of negative self-concept. On the other hand, scale developers must be cautious in their use of CIIAs, as some items that use contextual cues may contribute to the emergence of hidden framings. For example, items using “job” or “work” may prime work contexts (Schulze et al., 2021).

Much can still be learned about the optimal use of CIIAs in specific situations. For instance, it is likely that some CIIAs could improve the validity of personality scales when used in experimental research, but not when used in personnel selection. Further, little research has quantified the relative value of each CIIA. If a standard measure of personality were rewritten to include certain CIIAs, how much additional variance in “true personality” would the inclusion of each CIIA allow the scale to predict? This new, unified taxonomy of item attributes allows focused, cogent research in this area. Also, it encourages the identification and inclusion of new CIIAs as well as the recategorization of the current CIIAs ones to further refine and optimize our understanding of effective item design.
